# Allelic Lineages of the Ficolin Genes (*FCNs*) Are Passed from Ancestral to Descendant Primates

**DOI:** 10.1371/journal.pone.0028187

**Published:** 2011-12-15

**Authors:** Tina Hummelshøj, Janna Nissen, Lea Munthe-Fog, Claus Koch, Mads Frost Bertelsen, Peter Garred

**Affiliations:** 1 Laboratory of Molecular Medicine, Department of Clinical Immunology, Rigshospitalet, Faculty of Health Sciences, University of Copenhagen, Copenhagen, Denmark; 2 Department of Cancer and Inflammation, Institute of Molecular Medicine, University of Southern Denmark, Odense, Denmark; 3 Center for Zoo and Wild Animal Health, Copenhagen Zoo, Frederiksberg, Denmark; University of California Merced, United States of America

## Abstract

The ficolins recognize carbohydrates and acetylated compounds on microorganisms and dying host cells and are able to activate the lectin pathway of the complement system. In humans, three ficolin genes have been identified: *FCN1*, *FCN2* and *FCN3*, which encode ficolin-1, ficolin-2 and ficolin-3, respectively. Rodents have only two ficolins designated ficolin-A and ficolin-B that are closely related to human ficolin-1, while the rodent *FCN3* orthologue is a pseudogene. Ficolin-2 and ficolin-3 have so far only been observed in humans. Thus, we performed a systematic investigation of the *FCN* genes in non-human primates. The exons and intron-exon boundaries of the *FCN1-3* genes were sequenced in the following primate species: chimpanzee, gorilla, orangutan, rhesus macaque, cynomolgus macaque, baboon and common marmoset. We found that the exon organisation of the *FCN* genes was very similar between all the non-human primates and the human *FCN* genes. Several variations in the *FCN* genes were found in more than one primate specie suggesting that they were carried from one species to another including humans. The amino acid diversity of the ficolins among human and non-human primate species was estimated by calculating the Shannon entropy revealing that all three proteins are generally highly conserved. Ficolin-1 and ficolin-2 showed the highest diversity, whereas ficolin-3 was more conserved. Ficolin-2 and ficolin-3 were present in non-human primate sera with the same characteristic oligomeric structures as seen in human serum. Taken together all the *FCN* genes show the same characteristics in lower and higher primates. The existence of trans-species polymorphisms suggests that different *FCN* allelic lineages may be passed from ancestral to descendant species.

## Introduction

The ficolins are pattern recognition molecules of importance for innate immunity [1]. Their key function is to bind carbohydrates and acetylated compounds on microorganisms and dying host cells thereby enabling activation of the lectin pathway of the complement system through MBL/ficolin-associated serine proteases (MASPs) [1]. This complex formation mediates clearance of infectious agents and cellular debris by complement dependent phagocytosis. The ficolins have been shown to bind a plethora of bacteria such as *S. aureus*, *S. typhiurium*, *E. coli* and *A. viridans* [1]. In humans three ficolins have been identified: ficolin-1 (M-ficolin), ficolin-2 (L-ficolin) and ficolin-3 (H-ficolin), which are derived from the *FCN1*, *FCN2* and *FCN3* genes, respectively. Ficolin-1 and ficolin-2 are 80% homologous at the amino acid level, whereas ficolin-3 is approximately 50% homologous to ficolin-1 and ficolin-2.

Ficolin-1 is primarily expressed by monocytes, granulocytes and by myeloid progenitor cells in the bone marrow, but a minor expression of ficolin-1 is also observed in the spleen and in the lung [2]–[4]. Ficolin-1 was originally considered a non-serum protein that is secreted from cells and exerts its function locally in tissues [5]. It has recently been shown that ficolin-1 is present in low concentrations in human serum [6], [7]. However, the exact concentration is still a matter of debate. Ficolin-2 is predominantly expressed in the liver and it is found in human serum with a mean concentration of around 5 µg/ml [8]. Several single nucleotide polymorphisms (SNPs) in the promoter region of the *FCN2* gene have been shown to regulate the serum concentration of ficolin-2 and polymorphisms in the coding regions affect the binding of ficolin-2 to ligands [8], [9]. Ficolin-3 is highly expressed in lung and liver, but lower expression is also observed in heart, kidney, spleen, pancreas and placenta [10]. Ficolin-3 expression in the lung exceeds the expression in the liver, suggesting an important role in the lung. Ficolin-3 is present in human serum with a mean concentration of around 25 µg/ml [11].

In humans the *FCN1* and *FCN2* genes are located back-to-back on chromosome 9q34 and *FCN3* is located on chromosome 1p36.11. *FCN1* contains nine exons, whereas *FCN2* and *FCN3* each contain eight exons. The exon organisation of *FCN1* and *FCN2* resemble each other, although *FCN1* contains an extra exon, encoding an additional segment of four Gly-Xaa-Yaa repeats (where Xaa and Yaa represent any amino acid) [12]. The *FCN* genes encode similar polypeptide chains containing an N-terminal region rich in cysteine residues, a collagen-like domain with repeats of Gly-Xaa-Yaa segments of varying length, and a linker region followed by the fibrinogen-like (FBG) domain. The collagen-like domain interacts with the MASPs and activates the lectin complement pathway [1]. The FBG domain enables the ficolins to bind to ligands [13]. Three identical polypeptide chains of 34–35 kDa are assembled into structural subunits through the collagen-like domain and these subunits are further assembled into higher oligomeric structures increasing ligand avidity [1].

The *FCN* genes have been identified in a number of species such as mice, rats, chickens, pigs, hedgehogs, frogs and in the invertebrate ascidians. Rodents express only two ficolins designated ficolin-A and ficolin-B. Recently, it was suggested that the mouse *fcnb* gene is the orthologue to the human *FCN1* gene whereas the mouse *fcna* gene more likely seems to be a paralogue to the mouse *fcnb* gene, as the human *FCN2* gene is the paralogue to the human *FCN1* gene [14], [15]. Searches in databases and phylogenetic tree analyses have demonstrated that ficolin precursor molecules have gone through expansions involving independent duplication events in the different branches of the evolutionary tree [15], [16].

Phylogenetic analysis suggests that the mammal *FCN3* gene branched out by gene duplication in early vertebrate evolution [15], [16]. Based on this, the origin of ficolin-3 goes back to the evolutionary stage before the divergence of frogs. Nevertheless, in mice and rats the *FCN3* gene is found as a pseudogene containing several stop codons [14]. So far, no orthologue to the human ficolin-2 or ficolin-3 molecules has been described in other species.

Here we describe the characterization of the *FCN* genes in a number of non-human primate species and demonstrate for the first time the ficolin-2 and ficolin-3 structure in sera outside the human situation in different primate species. Moreover, we provide strong evidence that allelic lineages of the *FCN* genes may have been past during evolution.

## Results

### The primate FCN genes

**Figure 1:**
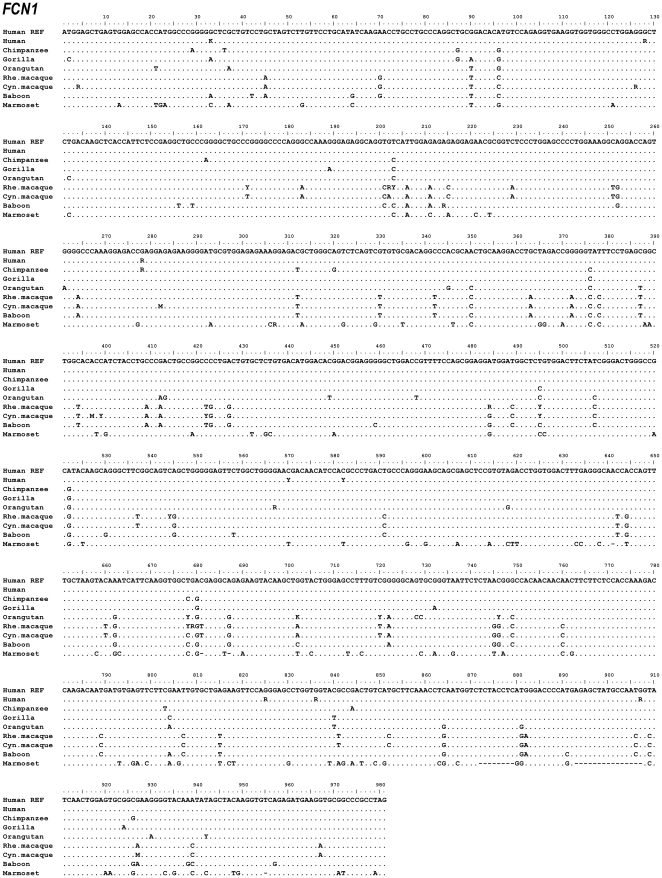
Alignment of the *FCN1* nucleotide sequences. Hum REF is the human reference sequence containing the major alleles. Gene variation among a species is given by R (A or G), Y (C or T), K(G or T), M (A or C), S (G or C) or W (A or T).

**Figure 2:**
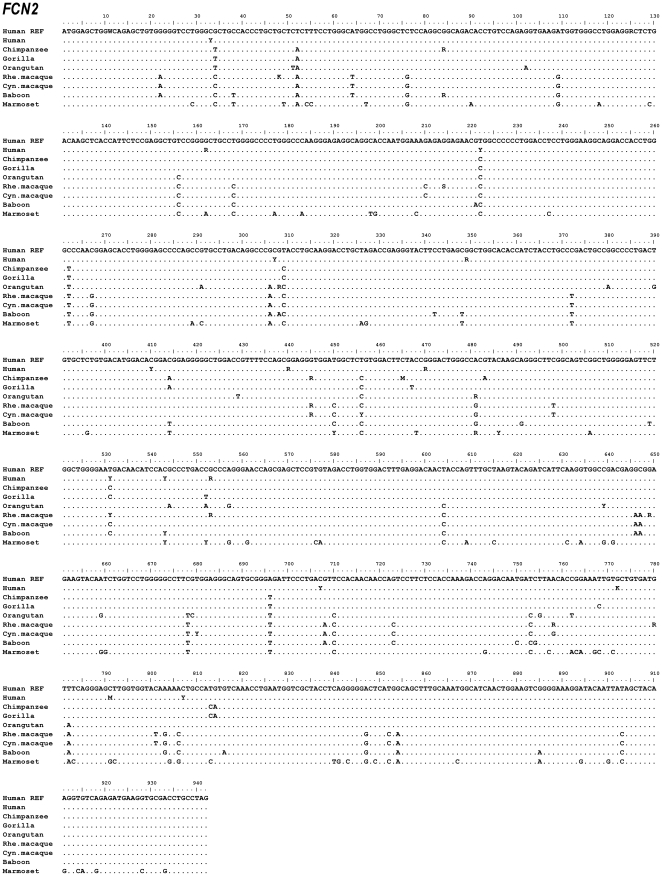
Alignment of the *FCN2* nucleotide sequences. Hum REF is the human reference sequence containing the major alleles. Gene variation among a species is given by R (A or G), Y (C or T), K(G or T), M (A or C), S (G or C) or W (A or T).

**Figure 3:**
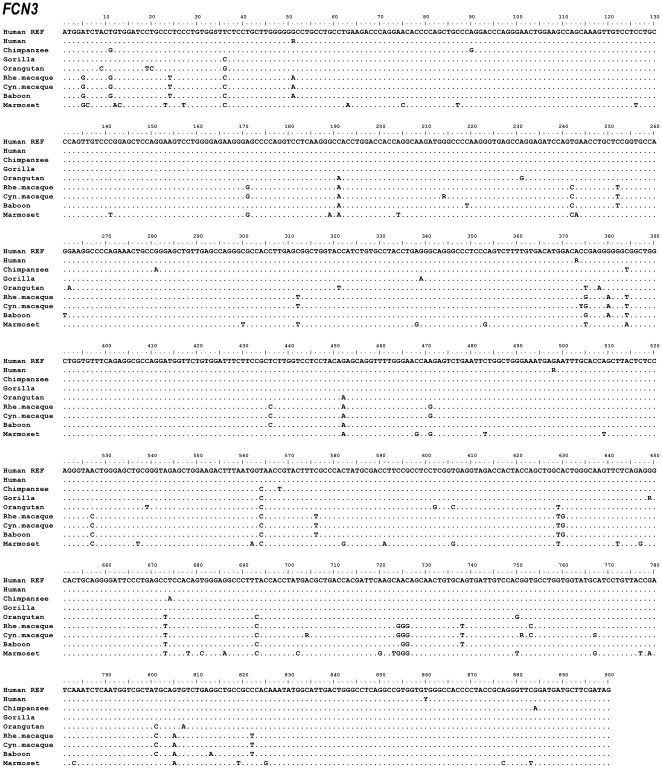
Alignment of the *FCN3* nucleotide sequences. Hum REF is the human reference sequence containing the major alleles. Gene variation among a species is given by R (A or G), Y (C or T), K(G or T), M (A or C), S (G or C) or W (A or T).

**Figure 4:**
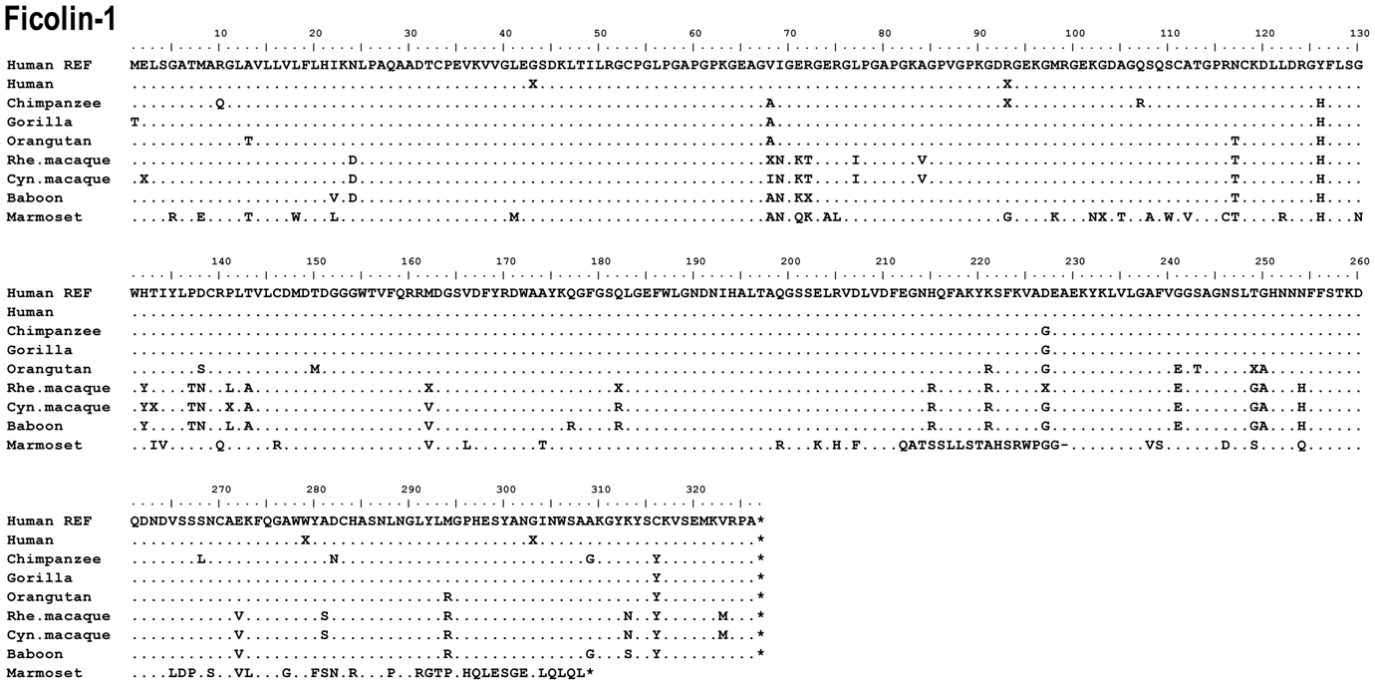
Alignment of the ficolin-1 protein sequences. Hum REF is the human reference sequence containing the major alleles. Gene variation among a species is given by an X.

**Figure 5:**
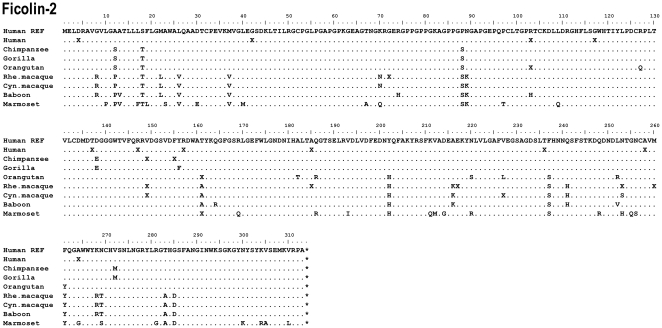
Alignment of the ficolin-2 protein sequences. Hum REF is the human reference sequence containing the major alleles. Gene variation among a species is given by an X.

**Figure 6:**
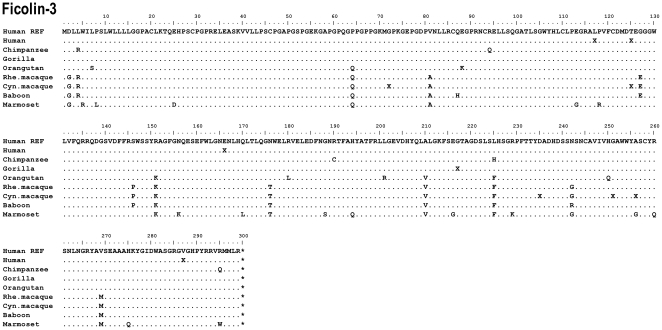
Alignment of the ficolin-3 protein sequences. Hum REF is the human reference sequence containing the major alleles. Gene variation among a species is given by an X.

The exons and intron-exon boundaries of the *FCN1*, *FCN2* and *FCN3* genes were sequenced on genomic DNA from the following higher and lower primate species: chimpanzee, gorilla, orangutan, rhesus macaque, cynomolgus macaque, baboon and common marmoset. Alignment data of *FCN1*, *FCN2* and *FCN3* nucleotide sequences are shown in figures 1, 2, 3, respectively. Alignment data of ficolin-1, ficolin-2 and ficolin-3 protein sequences are shown in figures 4, 5, 6, respectively. Positions of all the DNA variations among each species are counted in relation to the human *FCN1*, *FCN2* or *FCN3* translation start site with the A nucleotide of ATG being nucleotide position (nt) +1. Data of discovered variations are given in table 1A (ficolin-1), table 2A (ficolin-2) and table 3A (ficolin-3). All these variations were observed in more than one individual or observed as heterozygote sequences indicating a true genetic variation. Deviations from the published reference sequences are given in tables 1, 2, 3B. In these nucleotide positions we could not confirm the allele given by the gene databases in any of the investigated individuals.

**Table 1:** Variation in the *FCN1* gene.

(A)	*SS number*	*Nt pos*	*H-ref*	*P-ref*	*Var*	*Exon*	*AA change*
Cynomolgus macaque	410999239	+4	G	G	A	1	**Glu2Lys**
Cynomolgus macaque	410999243	+1433	G	G	A	2	Glu42Glu
Rhesus macaque	410999810	+1478	C	C	T	2	Pro57Pro
Rhesus macaque	410999812	+1509	G	A	G	2	**Ile68Val**
Rhesus macaque	410999815	+1510	T	T	C	2	**Ile68Ala**
Baboon	411633418	+1521	A	A	G	2	**Arg72Gly**
Chimpanzee	410998762	+3458	G	G	A	4	**Arg93Gln**
Cynomolgus macaque	410999245	+3462	A	A	C	4	Gly94Gly
Marmoset	411633438	+3487	G	G	A	4	**Gly103Arg**
Cynomolgus macaque	410999247	+4785	A	A	C	6	**Thr133Pro**
Cynomolgus macaque	410999249	+4787	C	C	T	6	Thr133Thr
Cynomolgus macaque	410999251	+4810	C	T	C	6	**Leu141Pro**
Rhesus macaque	410999817	+5272	A	A	G	7	**Met162Val**
Rhesus macaque	410999819	+5283	T	C	T	7	Ser165Ser
Cynomolgus macaque	410999252	+5283	T	C	T	7	Ser165Ser
Rhesus macaque	410999821	+5332	C	C	T	7	**Arg182Trp**
Orangutan	411000703	+5355	G	G	A	7	Gly189Gly
Orangutan	411000733	+6684	T	T	C	8	Ala226Ala
Rhesus macaque	410999822	+6684	T	C	T	8	Ala226Ala
Rhesus macaque	410999824	+6685	G	G	A	8	**Gly227Ser**
Orangutan	411633412	+6708	T	T	G	8	Leu234Leu
Orangutan	411000734	+6726	C	C	T	8	Val240Val
Orangutan	411000736	+7839	C	C	T	9	**Thr249Met**
Cynomolgus macaque	410999254	+8020	G	A	C	9	Ala309Ala
Orangutan	411000738	+8035	T	C	T	9	Tyr314Tyr

(B)	*SS number*	*Nt pos*	*H-ref*	*P-ref*	*Var*	*Exon*	*AA change*
Gorilla	410999518	+2	T	N	C	1	**Met1Thr**
Orangutan	411000741	+1451	C	dC	C	2	Thr48Thr
Orangutan	411000743	+1472	G	dG	G	2	Gly55Gly
Marmoset	411633439	+6650	A	G	T	8	**Ala215Ser**

***SS:*** *NCBI submission number,* ***Nt pos*** *: nucleotide position,* ***H-ref*** *: human reference sequence,* ***P-ref*** *: primate reference sequence,*

***Var*** *: variation detected,* ***AA change*** *: amino acid change. The non-synonymous variations are highlighted in bold.*

(A) Genetic variations identified in the *FCN1* gene. (B) Disagreement with the reference sequence in the *FCN1* gene.

**Table 2:** Variation in the *FCN2* gene.

(A)	*SS number*	*Nt pos*	*H-ref*	*P-ref*	*Var*	*Exon*	*AA change*
Rhesus Macaque	411021567	+48	G	G	T	1	Leu16Leu
Chimpanzee	411018865	+84	G	G	A	1	Ala28Ala
Baboon	411633428	+84	G	G	A	1	Ala28Ala
Rhesus Macaque	411021569	+1818	G	G	C	2	**Gly72Arg**
Orangutan	411020623	+4424	G	G	A	5	**Arg103His**
Chimpanzee	411018867	+4962	G	G	A	6	**Val149Met**
Rhesus Macaque	411021571	+4962	G	G	A	6	**Val149Met**
Cytomolgus Macaque	411019266	+4962	G	G	A	6	**Val149Met**
Marmoset	411633440	+4967	T	C	T	6	Asp150Asp
Cytomolgus Macaque	411019268	+4973	T	C	T	6	Ser152Ser
Chimpanzee	411018869	+4982	C	C	A	6	**Phe155Leu**
Orangutan	411020624	+4998	A	A	G	6	**Thr161Ala**
Marmoset	411633441	+4998	A	G	A	6	**Ala161Thr**
Marmoset	411633442	+5003	C	C	T	6	Tyr162Tyr
Rhesus Macaque	411021573	+5048	T	C	T	6	Asn177Asn
Baboon	411633429	+5060	C	C	T	6	His181His
Marmoset	411633443	+5060	C	C	T	6	His181His
Marmoset	411633444	+5069	C	C	T	6	Thr184Thr
Rhesus Macaque	411021575	+5070	G	G	A	6	**Ala185Thr**
Orangutan	411020627	+5688	C	C	T	7	Ala213Ala
Rhesus Macaque	411021577	+5698	G	G	A	7	**Glu217Lys**
Cytomolgus Macaque	411019269	+5729	T	T	C	7	**Val227Ala**
Rhesus Macaque	411021581	+6410	A	G	A	8	**Ser253Asn**
Rhesus Macaque	411021583	+6432	G	G	A	8	**Met260Ile**

(B)	*SS number*	*Nt pos*	*H-ref*	*P-ref*	*Var*	*Exon*	*AA change*
Rhesus Macaque	411021584	+84	G	A	G	1	Ala28Ala
Chimpanzee	411018872	+3940	C	T	C	4	**Leu97Pro**
Rhesus Macaque	411021586	+4947	G	A	G	6	**Ile144Val**
Gorilla	411019539	+4962	G	N	G	6	**Val149Val**
Orangutan	411020629	+5023	G	A	G	6	**Gln169Arg**
Marmoset	411633445	+5045	G	A	G	6	Gly176Gly
Marmoset	411633446	+5073	C	A	C	6	Arg186Arg
Marmoset	411633447	+5644–5645	-	G	-	7	Asp200Asp
Orangutan	411020631	+5697	G	A	G	7	Ala216Ala
Chimpanzee	411018874	+6478	A	G	A	8	Asp276Asn

***SS:*** *NCBI submission number,* ***Nt pos*** *: nucleotide position,* ***H-ref*** *: human reference sequence,* ***P-ref*** *: primate reference sequence,*

***Var*** *: variation detected,* ***AA change*** *: amino acid change. The non-synonymous variations are highlighted in bold.*

(A) Genetic variations identified in the *FCN2* gene. (B) Disagreement with the reference sequence in the *FCN2* gene.

**Table 3:** Variation in the *FCN3* gene.

(A)	*SS number*	*Nt pos*	*H-ref*	*P-ref*	*Var*	*Exon*	*AA change*
Cynomolgus Macaque	411021217	+836	G	G	A	3	**Gly72Ser**
Cynomolgus Macaque	411021219	+1664	C	C	T	5	**Thr125Met**
Gorilla	411021453	+4214	A	G	A	7	**Gly217Ser**
Cynomolgus Macaque	411021221	+5387	G	A	G	8	**Asp235Gly**
Cynomolgus Macaque	411021223	+5434	G	G	A	8	**Gly251Ser**
Cynomolgus Macaque	411021225	+5450	C	C	G	8	**Ala256Gly**

(B)	*SS number*	*Nt pos*	*H-ref*	*P-ref*	*Var*	*Exon*	*AA change*
Chimpanzee	411020913	+1606	T	G	T	5	**Gly106Trp**
Rhesus Macaque	410997753	+1647	C	T	C	5	**Val119Val**

***SS:*** *NCBI submission number,* ***Nt pos*** *: nucleotide position,* ***H-ref*** *: human reference sequence,* ***P-ref*** *: primate reference sequence,*

***Var*** *: variation detected,* ***AA change*** *: amino acid change. The non-synonymous variations are highlighted in bold.*

(A) Genetic variations identified in the *FCN3* gene. (B) Disagreement with the reference sequence in the *FCN3* gene.

### Gene variations in FCN1

A total of 25 DNA variations were found in the coding region of which twelve resulted in an amino acid substitution (table 1A). These non-synonymous variations were found in chimpanzee: Arg93Gln; orangutan: Thr249Met; rhesus macaque: Ile68Val, Ile68Ala, Met162Val, Arg182Trp, Gly227Ser; cynomolgus macaque: Gly2Lys, Thr133Pro, Leu141Pro; baboon: Arg72Gly and common marmoset: Gly103Arg. Interestingly, the non-synonymous variation Arg93Gln found in chimpanzee has previously been described in the human *FCN1* gene (rs56345770).

In contrast to the human polymorphism +33G>T/Gly11Gly (rs10858293) different alleles were found in both the gorilla (+33A), the baboon (+33A) and in the common marmoset (+33C) (figure 1), however all the alleles code for the same amino acid, glycine.

A few disagreements with the reference sequence were observed in some of the investigated primate species (table 1B). The reference sequence of the gorilla defines the nucleotide at nt+2 in exon 1 to be any base (A, T, G or C). In this study the nucleotide was determined to be a C nucleotide. Compared to the human gene, this nucleotide is a part of the initiating codon ATG coding for the amino acid methionine. Thereby the gorilla does not have the same start site in nt+1–3 since this codon is changed from an ATG to an ACG. Instead another initiating site is located in codon 8 making the gorilla ficolin-1 polypeptide seven amino acids shorter in the N-terminal region compared to the human polypeptide.

**Figure 7:**
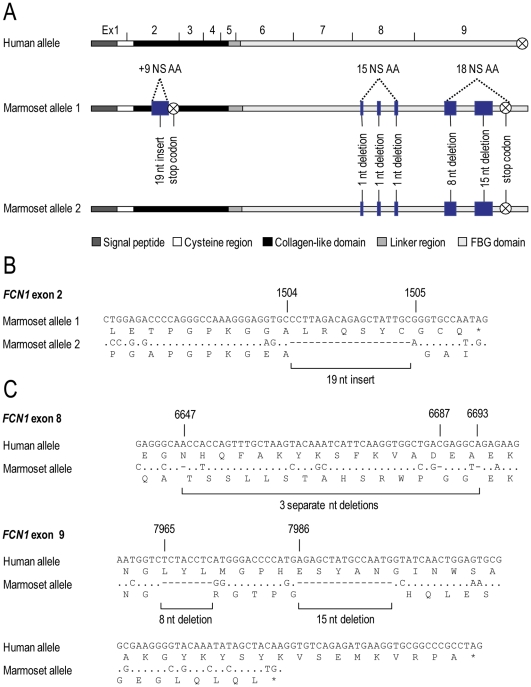
Illustration of the domain organisation of the human and marmoset *FCN1* genes (A). Two alleles of marmosets *FCN1* were identified. Exons are indicated with black vertical lines. Variations are indicated with blue boxes. Ex: exon, NS AA: non-synonymous amino acid, nt: nucleotide. Alignment of nucleotide/protein sequences of exon 2 of the two *FCN1* marmoset alleles (B). The marmoset allele 1 sequence has an insertion of 19 nucleotides between nt+1504–1505, which causes a reading frame shift, and subsequently gives rise to a premature stop codon in nt +1514. Alignment of nucleotide/protein sequences of exon 8 and exon 9 of the human and marmoset *FCN1* genes (C). In exon 8 a nucleotide is deleted in nt +6647, which makes the sequence go out of reading frame. In nt +6687 and +6693 a nucleotide is deleted making the sequence go back into reading frame. These deletions change 15 amino acids in the FBG domain before it goes into reading frame again. In exon 9 eight nucleotides are deleted in nt +7965–7972 and moreover 15 nucleotides are deleted in nt +7986–8000, which causes a reading frame shift and give rise to a premature stop codon, making the ficolin-1 polypeptide ten amino acid shorter in the FBG domain than the human polypeptide.

Most of the investigated primate species showed identical *FCN1* gene structure, however dramatic differences were observed in the common marmoset *FCN1* gene.. The common marmoset has at least two different *FCN1* alleles (figure 7A). Allele 2 is similar to the human and other primates in the region of exon 2 whereas the allele 1 has an insert of 19 nucleotides between the human nt+1504 and +1505 (figure 7B). This insertion causes a reading frame shift, which further causes a premature AMB-stop codon to arise in nt+1514. Furthermore, in exon 8 a nucleotide (nt+6647) is deleted in both alleles of the common marmoset *FCN1* gene, which make the amino acid sequence shift in the reading frame compared to the human gene (figure 7C). However, two downstream deletions at nt+6687 and +6693 revert the sequence into correct reading frame. In total these three variations alter 15 amino acids of the FBG domain. Additionally, eight nucleotides are deleted in the N-terminal region of exon 9 (nt+7965–7972) and 15 nucleotides are deleted in the C-terminal region of exon 9 (nt+7986–8000) (figure 7C). This alters the reading frame and gives rise to a premature OPA-stop codon.

### Gene variations in FCN2

A total of 19 DNA variations were identified in the coding region of *FCN2* whereof ten were found in exon 6 (table 2A). Several non-synonymous variations were found in chimpanzee: Val149Met, Phe155Leu; orangutan: Arg103His, Thr161Ala; rhesus macaque: Gly72Arg, Val149Met, Ala185Thr, Glu217Lys, Ser253Asn, Met260Ile; cynomolgus macaque: Val149Met, Val227Ala and common marmoset: Thr161Ala. Of these the Ala185Thr variation in exon 6 of the rhesus macaque has previously been described in humans (rs55860122).

Several variations occurred in more than one primate specie, this included the variation in nt+84/Ala28Ala found in both the chimpanzee and the baboon; the variation in nt+4962/Val149Met found in the chimpanzee, the rhesus macaque and the cynomolgus macaque and finally the variation in nt+4998/Thr161Ala found in the orangutan and the common marmoset.

### Gene variations in FCN3

A total of six amino acid substituting variations were identified in the coding region of *FCN3* (table 3A). These non-synonymous variations were found in the gorilla: Gly217Ser and in the cynomolgus macaque: Gly72Ser, Thr125Met, Asp235Gly, Gly251Ser and Ala256Gly. The human *FCN3* gene variation, +51G>A/Gly17Gly (rs56088921), where G is the major allele in humans, is found as an A nucleotide in the rhesus macaque, the cynomolgus macaque and the baboon (figure 3). The other investigated primate species had a G nucleotide located in this position. Reference sequence disagreements were only observed in the chimpanzee and the rhesus macaque (table 3B).

### Phylogenetic analysis

**Figure 8:**
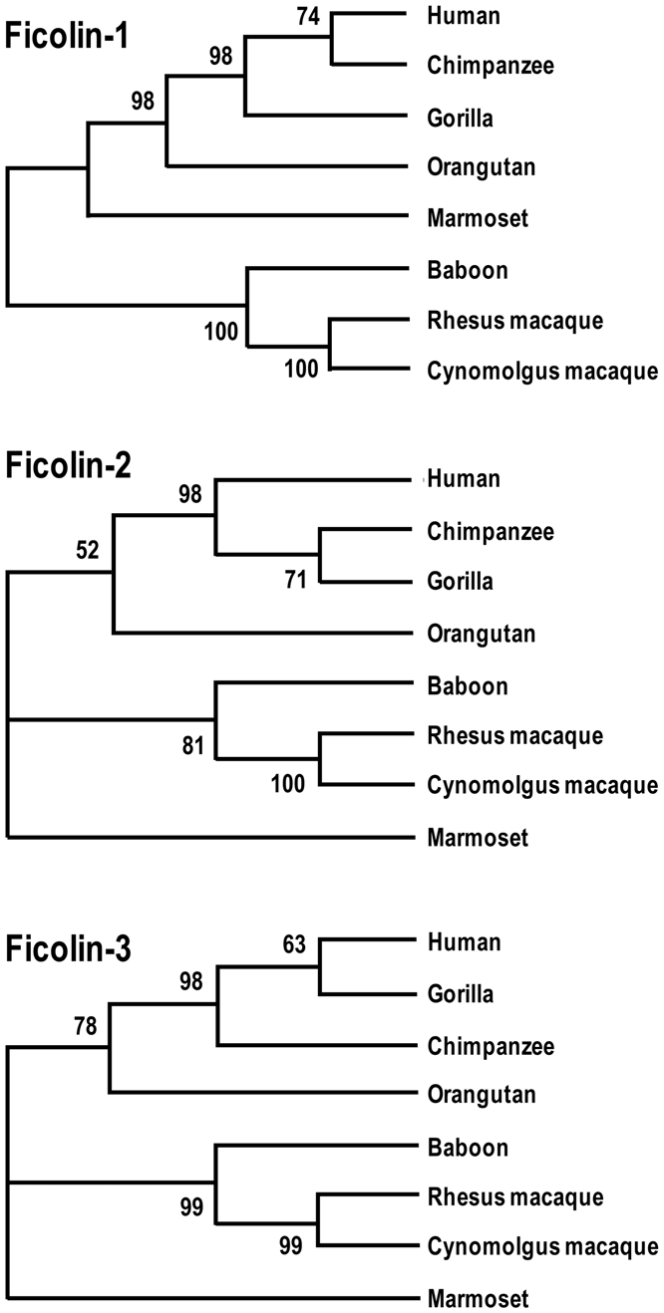
Phylogenetic relationship of the primate ficolins based on the proteins sequences. The neighbouring joining was bootstrapped 500 times. Based on several output files representative phylogenetic trees were constructed. Numbers on branches are bootstrap percentages supporting a given partitioning.

The entire amino acid sequences of the ficolins from all primates investigated were aligned and constructed into neighbouring joining trees. Based on several output files, representative phylogenetic trees were constructed. The phylogenetic trees reflect the expected relationship between human and primates (figure 8).

### Amino acid variability

**Figure 9:**
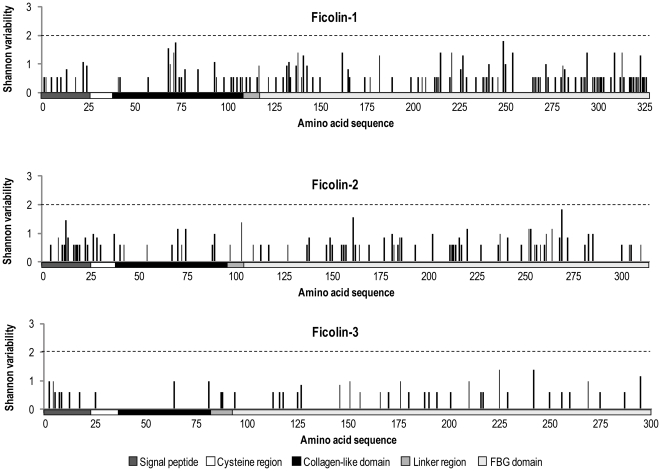
The amino acid diversity of the ficolins was evaluated by the Shannon entropy. The position of the functional domains of the ficolins is indicated below the x-axis. Values below 2 are considered as conserved residues and the value 0 represent that only one amino acid is present at that position.

The amino acid variability among the investigated species was estimated by calculating the Shannon entropy which is a mathematical tool to estimate variability [17]. Values below 2 are considered as conserved residues and the value 0 indicate that only one amino acid is represented at that position. The Shannon analysis revealed that the ficolins were generally highly conserved (figure 9) with mean entropy values of 0.254 for ficolin-1, 0.198 for ficolin-2 and 0.102 for ficolin-3.

### Oligomeric structure of primate ficolin-2 and ficolin-3

**Figure 10:**
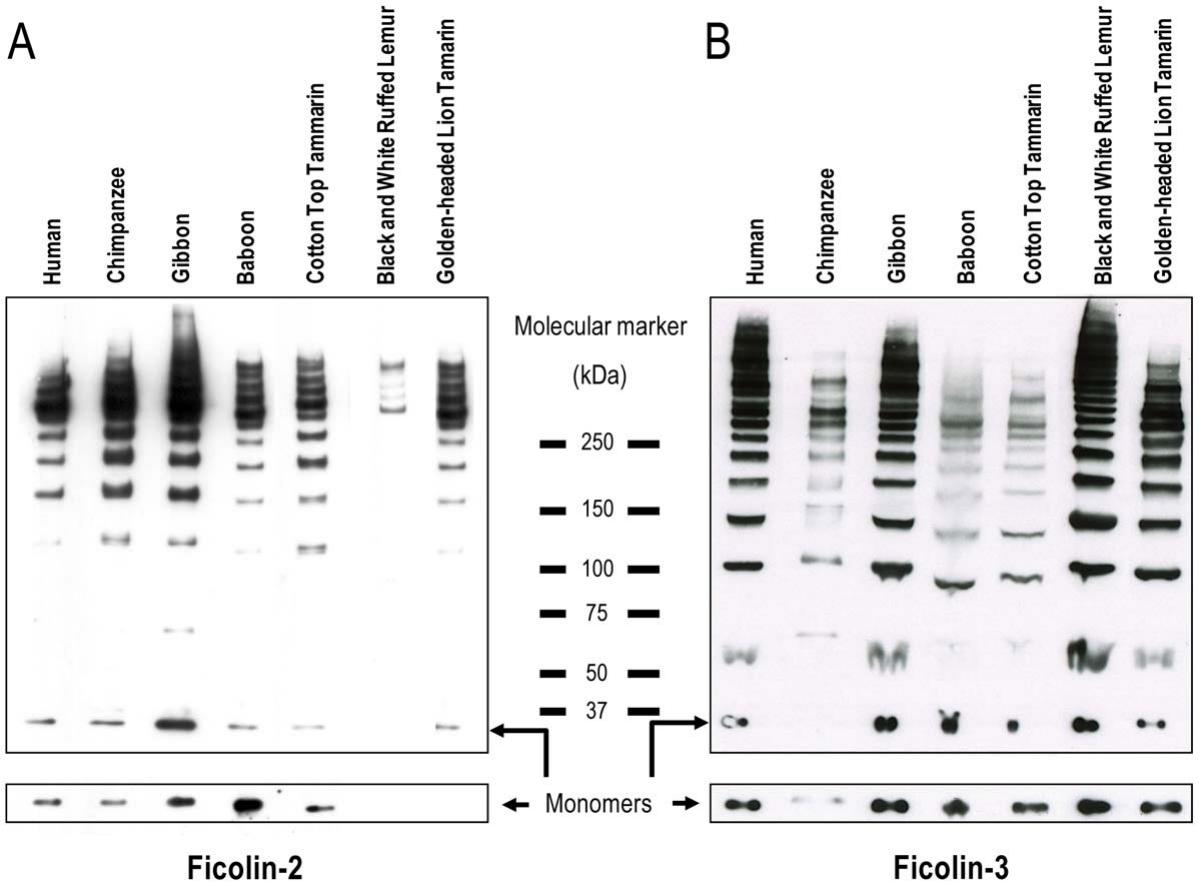
Oligomerization pattern of primate ficolin-2 and ficolin-3 was evaluated by SDS-PAGE subjected to western blot. Samples were analysed by SDS-PAGE on 3–8% Tris-actetate gels under non-reduced and reduced conditions subjected to western blot and detected with (A) mono- and polyclonal anti-ficolin-2 or (B) mono- and polyclonal anti-ficolin-3. Arrows show the multimers and monomers of the ficolins.

Serum ficolin-2 was detected in the following higher and lower primate species: chimpanzee, gibbon, baboon, cotton top tamarin, black and white ruffed lemur and golden-headed lion tamarin. The primates showed an identical banding pattern compared to the human protein at both reduced and non-reduced conditions (figure 10A). The monomer band for the black and white ruffled lemur and the golden-headed lion tamarin were not detected under reduced condition probably due to inadequately protein content or decreased cross-species specificity of the antibodies used for detection. Furthermore, we observed that some of the investigated chimpanzees had a weak extra band located just above the monomers, probably caused by different glycosylation of the monomers.

Serum ficolin-3 was detected in the following higher and lower primate species: chimpanzee, gibbon, baboon, cotton top tamarin, black and white ruffed lemur and black-handed spider monkey. The protein bands of the chimpanzee ficolin-3 was weak on western blot compared to the human and the other primates indicating that chimpanzees have a lower ficolin-3 serum concentration than all the investigated primate species. The banding pattern of serum ficolin-3 in higher and lower primate species was similar to human ficolin-3 (figure 10B). A slightly lower molecule weight of the oligomers was observed for the baboon and the cotton top tamarin.

### Ficolin-2 and ficolin-3 serum concentrations in chimpanzees

**Figure 11:**
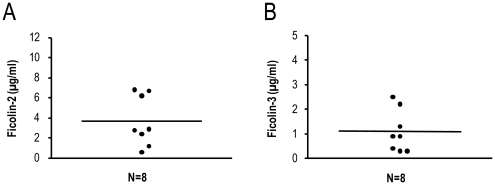
Individual serum concentrations of ficolin-2 (A) and ficolin-3 (B) were measured in eight chimpanzees. Horizontal lines indicate means.

In order to determine the serum concentrations of ficolin-2 and ficolin-3 in chimpanzees a sandwich ELISA was performed on serum from different chimpanzees using anti-human antibodies. Anti-ficolin-2 (FCN216/FCN219) or anti-ficolin-3 (FCN334/FCN334) monoclonal antibodies were used as coating and detection antibodies for ficolin-2 or ficolin-3, respectively. Whole serum was used as a source for ficolins. The ficolin-2 serum concentration was measured in serum from eight chimpanzees and ranged from 0.6–6.8 µg/ml with a mean serum concentration of 3.7 µg/ml (figure 11A). The ficolin-3 serum concentration was measured in serum from eight chimpanzees and ranged from 0.3–2.5 µg/ml with a mean serum concentration of 1.1 µg/ml (figure 11B).

## Discussion

This is the first report of the *FCN* genes in non-human primate species. The *FCN1*, *FCN2* and *FCN3* genes were investigated for DNA variations in samples from the following primate species: chimpanzee, gorilla, orangutan, rhesus macaque, cynomolgus macaque, baboon and common marmoset. As in humans, the *FCN* genes of the investigated primate species were found to be polymorphic and several novel variations in all three *FCN* genes within each primate species were found. Several variations compared with the reference sequences were observed in all three *FCN* genes which might reflect inter-individual variations.

**Figure 12:**
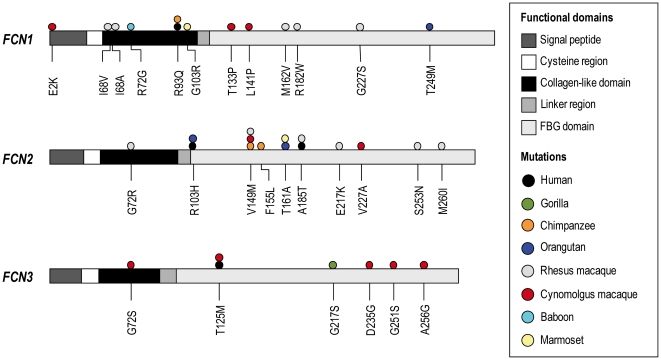
Location of the non-synonymous variants in the *FCN* genes. Illustration of the domain organisation of the *FCN1*, *FCN2* and *FCN3* genes. Genetic variations are indicated with coloured dots where each species has its own colour. If the variation has previously been described in human it is indicated with a black dot. The amino acid positions of the genetic variations are shown below the genes.

Non-synonymous variations in the coding region may be critical for the function and structure of the protein. They may introduce stop codons or frame shifts that lead to defective or truncated proteins. The post-translational modifications, intramolecular interactions and the three-dimensional structures could also be affected. When comparing the non-synonymous variations found in the human *FCN* genes with the primate *FCN* genes a striking similarity in the locations is observed (figure 12). Most of the non-synonymous variations are clustered in the FBG domain in the *FCN* genes and may thus affect ficolins affinity or specificity of carbohydrate binding to microorganisms. During evolution the FBG domain may have co-evolved with microorganisms and modification of the FBG domain could give rise to a broader diversity against different microorganisms.

Several non-synonymous variations were found in the collagen-like domain of *FCN1* in the primate species, which is not observed to the same degree in the *FCN2* or *FCN3* genes (figure 12). Furthermore, the Shannon entropy analysis indicates higher amino acid viability of ficolin-1 compared to the other ficolins. In the chimpanzee the non-synonymous *FCN1* variation Arg93Gln situated in exon 4 encoding the collagen-like region, has previous been described in human as a common variation with an allele frequency of 0.07 (rs56345770) in the Mozambique population and an allele frequency of 0.09 in the population from Ghana [18]. Furthermore, this variation was also detected in the Japanese population with a lower allele frequency of 0.01. Non-synonymous variation situated in the collagen-like domain may influence the interaction with the MASPs, which are responsible for activating the lectin pathway of the complement system. Whether these variations in the collagen-like domain affect the MASPs binding to ficolin-1 remain to be elucidated. These variations could also prevent the correct folding of the oligomers, causing protein instability, reduced protein concentration and/or reduced binding capacity towards ligands due to incomplete oligomerization.

In the signal peptide of *FCN1* a novel non-synonymous variation Glu2Lys was found in the cynomolgus macaque that might affect the secretion of the protein. In the FBG domain six new non-synonymous variations were detected (figure 12). Non-synonymous variation situated in the FBG domain may affect the affinity and specificity of the carbohydrate binding towards microorganisms. To elucidate the functional effect of these variations further molecular investigations are needed. The crystal structure of the FBG domain of ficolin-1 and it interaction with ligands have been investigated by Garlatti et al. [19]. In the chimpanzee, the residue Ser268 is found as a Leu268 (named Ser239 in the paper by Garlatti et al. [19] since their nomenclature does not contain the signal peptide of 29 amino acids). This amino acid is involved in the Ca^2+^ coordination of ficolin-1 and the ligand binding may thereby be affected in the chimpanzee. Furthermore, the Gly250 (Gly221 in Garlatti et al. [19]) situated in the S1 binding site are found as an alanine residue in the orangutan, the rhesus macaque, the cynomolgus macaque and the baboon. This may change the binding pocket and thus interfere with ligand binding.

The initiating codon methionine (ATG) of the gorilla *FCN1* gene was found to be located in residue 8 with respect to the human sequence. This variant affects the signal peptide by a reduction of 7 amino acids and whether the secretion of the protein is affected remains to be investigated.

A significantly different allele of the *FCN1* gene was detected in the common marmoset. This novel allele (named allele 1) carries an insert of 19 nucleotides resulting in an altered amino acid composition in the N-terminal region of the molecule spanning from nt+1504 to a premature stop codon in nt+1514 thus giving rise to a putative truncated form of ficolin-1. Individuals heterozygous for this variant allele may have a lower ficolin-1 concentration compared to individuals with the “normal” allele and homozygous individuals may likely have a significantly altered ficolin-1 molecule that even may be non-functional. Moreover, the FBG domain of both common marmoset *FCN1* alleles lack several nucleotides compared to the human sequence affecting 25 amino acids. The functional effect of these differences in the FGB domain of the common marmoset ficolin-1 molecule remains to be elucidated.

Several novel non-synonymous variations were found in the *FCN2* gene in the investigated primate species. The Ala185Thr variation detected in the rhesus macaque has previous been described in human in the Japanese population (rs55860122) with a frequency of 0.01 [18]. Interestingly, the non-synonymous variation Val149Met occurred in more than one primate species, namely in the chimpanzee, the rhesus macaque and the cynomolgus macaque. Additionally, the Thr161Ala was found in both the orangutan and marmoset. The crystal structure of the FBG domain of the ficolin-2 and its interactions with ligands has been described [20] showing that the amino acid Thr161 (Thr136 in Garlatti et al. [20] since their nomenclature does not contain the signal peptide of 25 amino acids) is located in the S3 binding site and that the amino acids is involved in the interaction with GalNAc, acetylcholine and curdlan. This variation, Thr161Ala, may likely affect the interaction with ligands.

The crystal structure of ficolin-2 also indicates that Van der Waals contact is established between Phe114 and Gln164 (Phe89 and Gln139 in Garlatti et al. [20]). The baboon contained an arginine in residue 164 instead of a glutamine and may interfere with the inter protomer interfaces of ficolin-2. Furthermore, the Asn253 (Asn228 in Garlatti et al. [20]) is involved in the Ca^2+^ coordination and the common marmoset contains a histidine in this residue, which might affect the interaction with Ca^2+^ and thereby affect the Ca^2+^-dependent ligand binding.

A synonymous *FCN2* variation +5060C>T/His181His was found in the baboon and the common marmoset. This variation was previously detected in the human samples from Argentina, Japan, Denmark and Ghana (rs34789496) with an allele frequency of 0.19, 0.14, 0.03 and 0.01, respectively [18].

Moreover, the human synonymous variation in nt+177G>A/Gly54Gly (rs55865317) is found as an A nucleotide in the common marmoset but not in the rest of the investigated primate species which has a G nucleotide in this position. This may indicate that the A to G nucleotide exchange may have arisen after the branching of the marmoset lineage.

An intact and highly conserved *FCN3* gene was found in all the investigated primate species. Nevertheless, in mice and rats the *FCN3* gene has been identified as a pseudogene and it is reasonable to assume that the gene-inactivation has arisen after the branching of the rodent lineages [14]–[16]. As for human, the primate *FCN3* genes contained only few genetic variations in contrast to the *FCN1* and *FCN2* genes. Only six different variations were found, which were distributed between the gorilla: Gly217Ser and the cynomolgus macaque: Gly72Ser, Thr125Met, Asp235Gly, Gly251Ser and Ala256Gly. Why the *FCN3* gene of the cynomolgus macaque was found to be more polymorphic than the rest of the investigated primates remains speculative. In human *FCN3*, a synonymous variation is described in nt+51G>A/Gly17Gly (rs55865317). This variation was not found as a mutation in any of the investigated species, however, the +51 nucleotide is found as an A in the rhesus macaque, the cynomolgus macaque and the baboon. The rest of the investigated primate species all had a G nucleotide located in this position, indicating that this variation has arisen after the branching of the primate lineages.

The phylogenetic relationship between the primate ficolins was analyzed by neighbouring joining trees created from the entire proteins sequences. For all tree ficolins, the sequences from human, gorilla and chimpanzee formed a tight cluster supported by high bootstrap percentages. Also the baboon, rhesus macaque and cynomolgus macaque sequences formed tight clusters. When analyzing the amino acid variability among the primate ficolins, the Shannon entropy indicated a general high conservation between the investigated species. However, ficolin-1 and ficolin-2 tend to be more variable than ficolin-3.

Few *FCN* polymorphisms were not exclusive for one species. This could be compatible with the notion that a number of founder individuals are carrying genetic information over the species barriers and that allelic lineages may be passed from ancestral to descendant species. This phenomenon is well described in the major histocompatibility complex system as so-called trans-species polymorphisms [21]. However, it cannot be excluded that our observation is due to convergent evolution although this does not seem to be very likely based on our phylogenetic tree analysis and nucleotide alignment data.

It was not possible to collect serum samples from all sequenced primate species. Nevertheless, we obtained serum samples from the following primate species: chimpanzee, gibbon, baboon, cotton top tamarin, golden-headed lion tamarin, black and white ruffled lemur and black-handed spider monkey. All the assays performed on primate serum were optimised on human serum using anti-human ficolin antibodies. However, the antibodies also reacted against primate ficolins, compatible with the sequence data that non-human primate and human ficolins are very homologous.

Ficolin-1 is found in very low concentration in human serum [6], [7]. Due to the low quantities of non-human primate serum available for this investigation and the lack of a reliable specific anti-ficolin-1 antibody we were not able to analyse the oligomeric structure and serum concentration of ficolin-1 in this study. However, all primate species investigated had detectable ficolin-2 protein in their sera when analyzing serum on SDS-PAGE followed by western blotting. The banding pattern of ficolin-2 was similar with the human ficolin-2 protein, under both reduced and non-reduced conditions. The ficolin-2 serum concentration was measured in serum samples from eight chimpanzees and was found in a mean concentration of 3.7 µg/ml. These findings are in agreement with the human ficolin-2 serum concentration with a mean concentration of 5.0 µg/ml [8]. In humans, the ficolin-2 concentration vary considerably among individuals and different polymorphisms in the promoter region have been described to decrease or increase the ficolin-2 concentration [9]. Whether the same is true for the chimpanzee remains to be established and will require both mapping of the promoter region of the *FCN2* gene as well as more chimpanzee individuals than those available for this study.

Until now, ficolin-3 has only been purified and characterized from humans. Serum samples from the primate species investigated were analysed on SDS-PAGE subjected to western blot. The investigated primate species all had detectable ficolin-3. All the primate species showed similar banding pattern when compared to human ficolin-3 protein under both reduced and non-reduced conditions. The ficolin-3 concentration in serum was analyzed in eight different chimpanzees and was found with a mean concentration of 1.1 µg/ml compared to a mean concentration of 25 µg/ml in the human situation [11]. The low ficolin-3 concentration observed in the chimpanzee could be a result of different circumstances. Although the chimpanzee *FCN3* gene is highly homologues to the human *FCN3* gene, having only four different amino acids (residue 94, 190, 225, and 295) in the mature protein the use of monoclonal antibodies may affect the ELISA results. However in SDS-PAGE/westen blot experiments, which also indicated a low ficolin-3 concentration, ficolin-3 was immunoprecipitated using a pool of anti-human-ficolin-3 antibodies and ficolin-3 was detected in western blot using a polyclonal anti-human-ficolin-3. Thus the mean ficolin-3 level in the investigated chimpanzees seems to be low when compared to humans. However, in the human situation the ficolin-3 level varies more than twenty fold from around 3 µg/ml to around 60 µg/ml among healthy individuals [11]. Thus, it could be that we detected a portion of the chimpanzees with relatively low ficolin-3 concentrations. The inter-individual variation in ficolin-3 concentration in humans cannot be accounted by any known regulatory variation in the promoter of human *FCN3* gene [11], but a rare deletion in exon 5 in the human *FCN3* gene decrease the serum concentration in heterozygous individuals with about 50% whereas homozygous individuals containing the deletion do not have any ficolin-3 protein [22]. Homozygosity for this deletion has been shown to be associated with immunodeficiency and severe necrotising enterocolitis in premature infants [22], [23]. This variation was not detected in the chimpanzees.

Taken together, this is the first time that ficolin-2 and ficolin-3 as proteins have been demonstrated outside the human species. Ficolin-2 and ficolin-3 from the investigated non-human primates showed the same characteristic oligomeric structure as seen in humans. Moreover, non-human primate *FCN* genes harbour polymorphisms that may be detrimental for the proteins in the homozygous situation. Finally a few polymorphisms were not specie specific, which suggests the existence of trans-species polymorphisms in the *FCN* system as has been documented in the past for the major histocompatibility complex system.

## Materials and Methods

### Primate samples

Primate DNA derived from two chimpanzees (*Pan troglodytes verus*), one gorilla (*Gorilla gorilla*), two orangutans (*Pongo pygmaeus*), four rhesus macaques (*Macaca mulatta*), four cynomolgus macaques (*Macaca fascicularis*), two baboons (*Papio hamadryas*) and two common marmosets (*Callithrix jacchus*) was obtained from commercially available American Tissue Typing Collection cell lines (Manassas, VA, USA).

Freshly drawn serum from chimpanzees (n = 8), white handed gibbon (n = 1), hamadryas baboon (n = 1), cotton top tamarin (n = 1), black and white ruffed lemur (n = 1) and black-handed spider monkey (n = 1), which were frozen at −80°C until testing, were obtained from the Center for Zoo and Wild Animal Health, Copenhagen Zoo, Copenhagen, Denmark.

### DNA sequencing of the FCN genes in primate species

Sequencing was performed as previously described [9]. Briefly, exons and intron-exon boundaries of the *FCN1*, *FCN2* and *FCN3* genes were sequenced using a single primer set, with a T7 tagged (5′-taatacgactcactataggg-3′) forward primer (DNA-Technology, Aarhus, Denmark) (table 4). Each PCR amplification reaction contained: 0.4 µl genomic DNA, 0.5 µM of each primer set, 1.7 mM MgCl_2_, 0.7 µM dNTP, and 0.25 U Platinum Taq DNA polymerase (Invitrogen, Taastrup, Denmark) at following cycling parameters: 2 min92°C, 30 cycles (30 s94°C, 30 s62°C, 60 s72°C), 5 min72°C. PCR products were sequenced using ABI BigDye cycle sequencing terminator kit (Applied Biosystems, Foster City, CA, USA) with 1 µM 5′ biotinylated T7 sequence primers. Following product purification with streptavidin sepharose beads (GE Healthcare, Brondby, Denmark) sequence analysis were performed on the ABI Prism 3130xl Genetic Analyzer (Applied Biosystems). The obtained DNA sequence was aligned using BioEdit software version 7.0.9.0 and DNA polymorphisms were visually confirmed by sequence electropherograms. Reference sequences for chimpanzee, orangutan, rhesus macaque and common marmoset were from the UCSC Genome Bioinformatics Site (http://genome.ucsc.edu) and reference sequence for the gorilla was from the Ensemble database (http://www.ensembl.org). No reference sequences were found for the cynomolgus macaque or the baboon. Thus we used the sequence of the rhesus macaque as reference. The following reference sequence were used: chimpanzee ficolin-1 (chr9:135043171–135051929), chimpanzee ficolin-2 (chr9:135013642–135020717), chimpanzee ficolin-3 (chr1:27605614–27612861), gorilla ficolin-1 (5995:42,607–52,194), gorilla ficolin-2 (5995:16,214–22,737), gorilla ficolin-3 (8428:57,060–62,627), orangutan ficolin-1 (chr9_random:3714–14502), orangutan ficolin-2 (chr9_random:12722926–12729662), orangutan ficolin-3 (chr1:202975217–202981008), rhesus ficolin-1 (chr15:3378237–3386312), rhesus ficolin-2 (chr15:3408984–3415856), rhesus ficolin-3 (chr1:30042434–30048405), common marmoset ficolin-1 (7611:78205–86501), common marmoset ficolin-2 (7611:45249–50085) and common marmoset ficolin-3 (1709:299350–303228).

**Table 4:** *FCN1*, *FCN2* and *FCN3* primers.

	Exon	Forward primer* (5′-3′)	Reverse primer (5′-3′)	Ch	Go	Or	Rm	Cm	Ba	Ma
*FCN1*	Exon 1	CTGTGGCACAAGGCGAGAG	CATCTTCACAGGAAGATGTGC	X	X				X	X
	Exon 1	GGAAACATCCTTTGAGATGACCTTTGAGATGACC	CGAGTGTCAGTGAGTGGGG			X	X	X		
	Exon 2	GACCAAGGCCCCAGCAG	TTGCCCAACTCTGCATCCA	X						
	Exon 2	GAAAACCTCACTTCCCAGCC	AGTTTCATAAAGAGCAAGAGCC		X	X	X	X	X	
	Exon 2	GAAAACCTCACTTCCCAGGC	TTGCCCAGCTCAGCATCTA							X
	Exon 3	TGGGGCAAAGATTTCCAGGG	CCAGGTCTCAATGGTGGG	X	X	X				
	Exon 3	CAGGACTTAACCACTTGGG	CCTGCAGGCTCACTAAGGC				X	X	X	X
	Exon 4	CCCACCATTGAGACCTGG	CCCACAGCCTGGATTGAC	X	X	X				
	Exon 4	CCACCATTGAGACCTGG	CCCACAGCCCAGATTGAC				X	X	X	
	Exon 4	CCCCACCCTTGAGATCTGG	GTCAGGGACATGGGCAGAC							X
	Exon 5	CTGACCAGGACAAAGGCTCT	GGATGATAGGTGCAGACAGG	X	X	X				
	Exon 5	GCTCTGATTTCAAGCTTATGAC	GGCCAACAGATGCAGACAAG				X	X	X	
	Exon 5	GCTTTGATTTTAAGCATATGAC	GGCCAACAGATGCAGACAAG							X
	Exon 6	GAGTCATGCTCGGTCTGC	5′TCACCCACCAGGTGTGCAGC	X						
	Exon 6	AGTCCTGCTCGGTCCGC	TCTACAACCAGGTGTGCAGC		X					
	Exon 6	GAAGCCTGTGGCCCTCC	AATAGAGAATTCCAGAGTGTG			X	X	X	X	
	Exon 6	CGAAGTCCTACTCTGTCCGC	GCAGCCTGGCCTCCAGG							X
	Exon 7	TGCTGTGGGACCTCGGCC	TTGCAGATGGCCCAGGCC	X	X					
	Exon 7	TACAAATGCTGCTCCTCTGG	TTGCAGATGGCCCAGGCC			X				
	Exon 7	TGCTGTGGGACGTCAGCC	GGTCCCAGTGCCACCCTG				X	X	X	
	Exon 7	GCTCCTCTGGAGGGCGGG-′3	CTAATGGGCAGGGGCACTTC							X
	Exon 8	CCCTCATGCCTGGTGACAG	CCAGGTTCTCTCTGCTTTCC	X	X	X	X	X	X	X
	Exon 9	CTTTTTCCAGCATCTGC	CATAATTCTCCCTCTGGTGAG	X	X	X	X	X	X	
	Exon 9	GGGCTCTTCCTAACATCTGC	CATAATTCTCCCTCTGGTGAG							X
*FCN2*	Exon 1	GAAATTGGAGTCTGAGGGAG	GGAAGCCACCAATCACGAAG	X	X	X	X	X	X	
	Exon 1	GGAAGATGAGAATTGGAGTCTG	CAGCTTTTGGGGGTGAGAAG							X
	Exon 2	AGATGCCTTTCAGTTGAGTGG	CTCGATCTAGGAACCATGGTG	X	X	X	X	X	X	
	Exon 2	GCAGCAGGTGCCTTTCAGTTGAG	CAGCAAGAGCCAGTGCCC							X
	Exon 3	AATGACAGCCGCCAGCTCC	GGCGTTGGCTCTGGCGAG	X	X	X				
	Exon 3	AATTACAGCCCCAGCTCAC	CCGCCCACATTCCTCTGC				X	X	X	
	Exon 3	GGCTGGGAGTTTGCTCAGG	CCTTGGCTGAAAGGACCTC							X
	Exon 4	GTCCGCGGACCAATGGGG	TCACTTCATTCTGGCAATGGC	X	X	X	X	X	X	X
	Exon 5	CCTACTGCCTGTGCCCTGC	GTGTGTTCTCCCACCAGGTG	X	X					
	Exon 5	CCCGCTGCCCCTGCCCTGC	GTGTGTTCTCCTGCCAGGTG			X			X	
	Exon 5	GCCTATGGCTCTGCTCCTC	CTCTGCCCAGGGCCCCTC				X	X		
	Exon 5	GCCTATGACCCTGGTCCTC	CCCTGCCCAGGACCCCTC							X
	Exon 6	TGTGGGACGTCGGCCTGG	AGAGGCTCTTGTGTTCCAGG	X	X	X				
	Exon 6	TGTGGGACGTCGGTCTGT	CAGCCCAGGCCTAAGGGG				X	X	X	
	Exon 6	TGTGGGACGTCGGCCTGG	CAGCCCAGGCCTAAGGGG							X
	Exon 7	CCATGTCTAAAGGTAGAGGGT	CACGCTCTCTCCACTTCCC	X		X	X	X	X	
	Exon 7	CCATGTCTAAAGGTAGAGAGC	CACGCTCTCTCCACTTCCC		X					
	Exon 7	TGCACGCACTGCTCCTCTGG	TTGCAGATGGCCCAGGCC							X
	Exon 8	CTGTCTGTAATGATGTTACTGC	TACAAACCGTAGGGCCAAGC	X	X	X	X	X	X	
	Exon 8	CTGCCTATAATGACATTACTGC	TATGAACCCAAGGGGTAAGC							X
*FCN3*	Exon 1	GTCCTCCCACCAGCCTG	GCAGAGCCCAGATTATGAAAC	X	X	X	X	X	X	X
	Exon 2	GTTTCATAATCTGGGCTCTGC	AAATTGCTACTTTCCTGCCTTC	X	X	X	X	X	X	
	Exon 2	GTTTCATAATCTGGGCTCTGC	AACTTGCTGCATTCTTGCCTTC							X
	Exon 3	CTCTGGCTCCAAGTCTCTTG	CCAAGCAGAGATCCCACCC	X	X	X	X	X	X	X
	Exon 4	CGGCTCCACTGGTGGCTC	CAACACCCAACACCATGTG	X						
	Exon 4	GAAGGATTCGGAAAAGGGCC	TGTGGGGAGGATCTTGGCC		X					
	Exon 4	TATGGAAGGCTGGGAGGAG	TGTGGGGAGGGGCTTGGCC			X				
	Exon 4	GAAGGATTTGGAAAAGAGCC	TGTGGGGAGGGGCTTGGCC				X	X	X	
	Exon 4	GACGGAAGGCTGGGAGGAG	TGTGGGGAGGGGCTTGGCC							X
	Exon 5	GGAATAACCTGGGCACCAC	CTCTGGTGGGTTCTGGCTC	X	X	X	X	X	X	
	Exon 5	GGAATAACCTGGTTGCCCC	GCCAACGGGGAGAAGTGAC							X
	Exon 6	CCTCACAGTTCCTGCATCC	CAGGATGGCAGACAGTAACC	X	X	X	X	X	X	X
	Exon 7	GGTTACTGTCTGCCATCCTG	ACAGAGGAGACAGGATTGCC	X	X	X	X	X	X	
	Exon 7	GGTTACTGTCTGCCCTCCTG	GCAGAGGAGAAGGGATTGCT							X
	Exon 8	ATTATATCTCCAAAGGTGCCAG	GGACAGGCAAGCAGAGGTG	X	X	X	X	X	X	X

Ch: chimpanzee, Go: gorilla, Or: orangutan, Rm: rhesus macaque, Cm: cynomolgus macaque, Ba: baboon, Ma: marmoset.

*containing a 5′ T7-sequence (5′-taatacgactcactataggg-′3).

### Phylogenetic analysis

The entire amino acid sequences of the ficolins from all primates investigated were aligned in the program BioEdit using ClustalW with default settings. The ficolin alignments were analyzed by the MEGA5 (M5b4) software to generate a neighbouring joining tree. The neighbouring joining was bootstrapped 500 times. Based on several output files representative phylogenetic trees were constructed.

### Amino acid variability

The amino acid variability was estimated by calculating the Shannon entropy which is a mathematical tool to estimate variability [24]. For a multiple protein sequence alignment, the Shannon entropy (H) for every position is as follow:
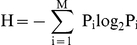



Where Pi is the fraction of residues of amino acid type i and M is the number of amino acid types (20). H ranges from 0 (if only one amino acid in present at that position) to 3 (if all amino acids are different in that position). Typically, positions with H>2.0 are considered variable, whereas those with H<2 are consider conserved. Highly conserved positions are those with H<1.0 [17]. Shannon values were calculated using the PVS (Protein Variability Server, Universidad Complutense de Madrid).

### The oligomeric structure of ficolin-2 and ficolin-3 in primate species

Serum ficolin-2 and ficolin-3 were immunoprecipitated using Pan Mouse Dynabeads (Invitrogen, Taastrup, Denmark) pre-incubated with mouse monoclonal anti-ficolin-2 antibodies (FCN216 and FCN219) or anti-ficolin-3 antibodies (FCN309 and FCN313). Following pre-incubation the beads were washed twice in 1× PBS with 0.05% Tween 20 (PBS-T) and serum samples were added and incubated at room temperature for 1 hour. After thoroughly washing of the beads with PBS-T the samples were diluted in LDS-buffer with or without reducing agent and heated for 5 minutes at 90°C. The samples were loaded on NuPAGE 3–8% Tris–acetate gels with Tris-Acetate SDS running buffer (Invitrogen, Taastrup, Denmark) and subsequently the separated proteins were blotted onto nitrocellulose membranes using the XCell II Mini-Cell blot apparatus, NuPAGE transfer buffer and Hybond ECL nitrocellulose membrane. Following blocking for 1 hour with 5% skim milk solution in PBS-T, ficolin-2 and ficolin-3 were detected using biotinylated antibodies (FCN219, FCN106 or FCN309, FCN313 and anti-hficolin-3 (BAF2367, R&D Systems, Abingdon, UK) respectively. After incubation with streptavidin-horseradish peroxidase (GE Healthcare, Brondby, Denmark) bands was visualized using Supersignal West Femto Maximum Sensitivity substrate (Pierce Biotechnology, Rockford, IL, USA).

### Determination of ficolin-2 and ficolin-3 serum concentration in chimpanzees

Ficolin-2 and ficolin-3 serum concentrations were measured in eight different chimpanzees using ficolin-2 and ficolin-3 specific ELISAs as previously described [8], [11]. Briefly, microtiter plates (Maxisorb, Nunc, Roskilde, Denmark) were coated with anti-ficolin-2 or anti-ficolin-3 monoclonal antibody (FCN216 or FCN334) in PBS. As calibration curve a normal human serum pool with known concentration of ficolin-2 and ficolin-3 was used. Samples were diluted 1∶25 and 1∶50 or 1∶64 and 1∶640 in triplicates in PBS-T and incubated three hours at 37°C. Bound ficolin were detected using biotinylated anti-ficolin-2 or ficolin-3 monoclonal antibody (FCN219 or FCN334) and subsequently Streptavidin-HRP (GE Healthcare, Brondby, Denmark). Plates were developed with OPD substrate solution containing H_2_O_2_ and terminated with 5 M H_2_SO_4_. The optical density was measured at OD 490 nm.

### Ethics

No specific permit was needed for this study, which was undertaken using banked serum obtained during routine work in the animal collection of Copenhagen Zoo. As part of the medical management of the collection, blood is drawn for chemistry panels and complete blood counts as well as serological screening in conjunction with internal and external animal transfers, as well as during clinical examinations. As part of this protocol –and in understanding of and compliance with Danish Law – an aliquot of serum is routinely banked for epidemiological follow-up or for studies such as this. The blood was drawn from anaesthetized animals and no additional venipuncture was required.
